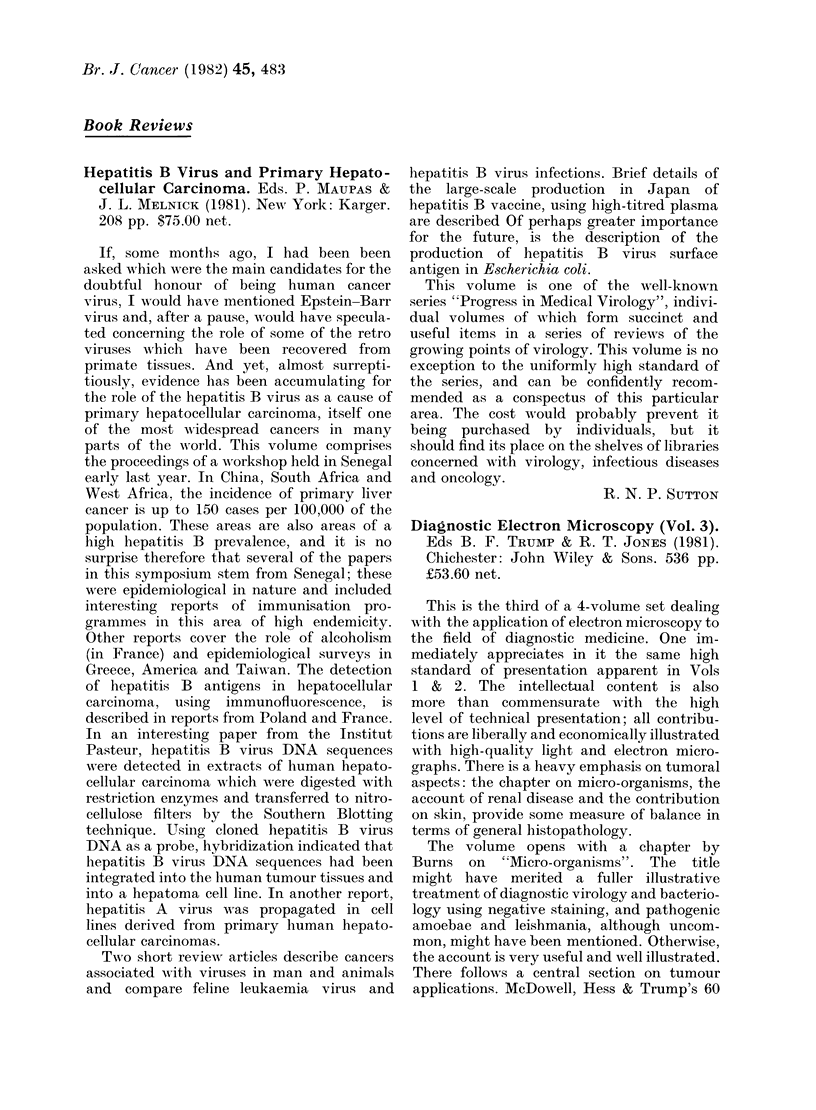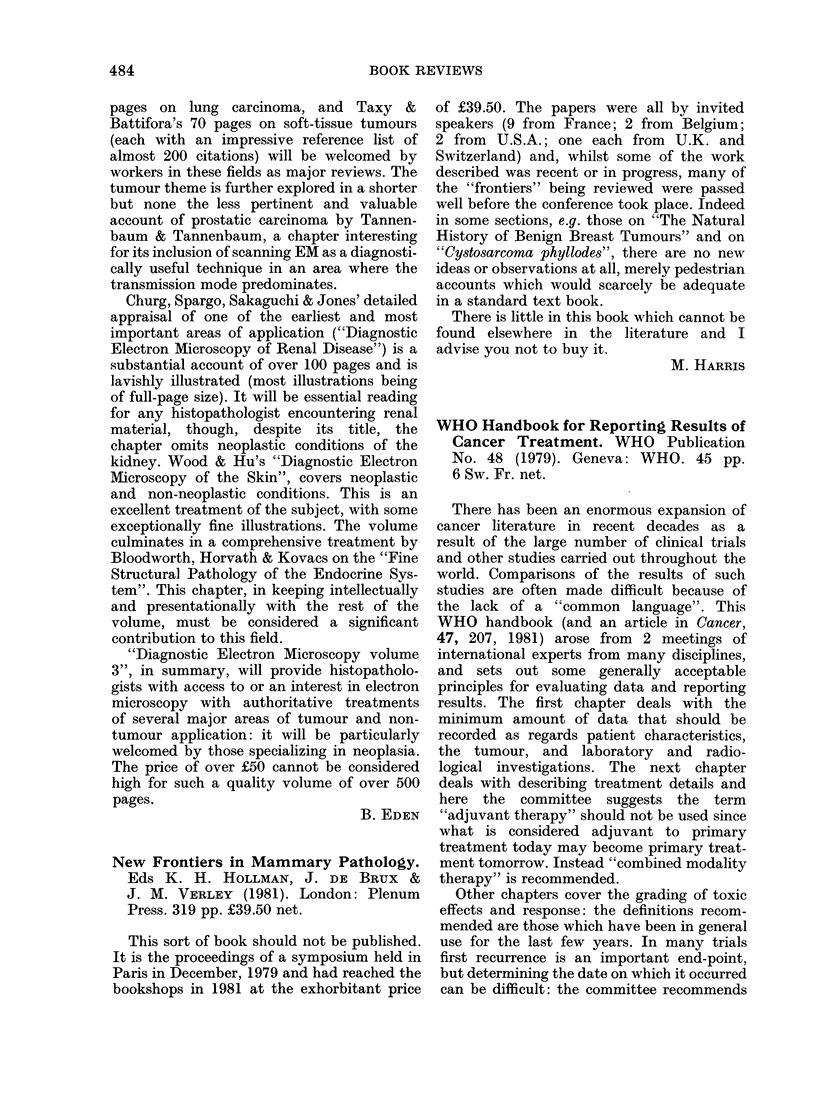# Diagnostic Electron Microscopy (Vol. 3)

**Published:** 1982-03

**Authors:** B. Eden


**Diagnostic Electron Microscopy (Vol. 3).** Eds B. F. TRUMP & R. T. JONES (1981). Chichester: John Wiley & Sons. 536 pp. £53.60 net.

This is the third of a 4-volume set dealing with the application of electron microscopy to the field of diagnostic medicine. One immediately appreciates in it the same high standard of presentation apparent in Vols 1 & 2. The intellectual content is also more than commensurate with the high level of technical presentation; all contributions are liberally and economically illustrated with high-quality light and electron micrographs. There is a heavy emphasis on tumoral aspects: the chapter on micro-organisms, the account of renal disease and the contribution on skin, provide some measure of balance in terms of general histopathology.

The volume opens with a chapter by Burns on "Micro-organisms". The title might have merited a fuller illustrative treatment of diagnostic virology and bacteriology using negative staining, and pathogenic amoebae and leishmania, although uncommon, might have been mentioned. Otherwise, the account is very useful and well illustrated. There follows a central section on tumour applications. McDowell, Hess & Trump's 60

pages on lung carcinoma, and Taxy & Battifora's 70 pages on soft-tissue tumours (each with an impressive reference list of almost 200 citations) will be welcomed by workers in these fields as major reviews. The tumour theme is further explored in a shorter but none the less pertinent and valuable account of prostatic carcinoma by Tannenbaum & Tannenbaum, a chapter interesting for its inclusion of scanning EM as a diagnostically useful technique in an area where the transmission mode predominates.

Churg, Spargo, Sakaguchi & Jones' detailed appraisal of one of the earliest and most important areas of application ("Diagnostic Electron Microscopy of Renal Disease") is a substantial account of over 100 pages and is lavishly illustrated (most illustrations being of full-page size). It will be essential reading for any histopathologist encountering renal material, though, despite its title, the chapter omits neoplastic conditions of the kidney. Wood & Hu's "Diagnostic Electron Microscopy of the Skin", covers neoplastic and non-neoplastic conditions. This is an excellent treatment of the subject, with some exceptionally fine illustrations. The volume culminates in a comprehensive treatment by Bloodworth, Horvath & Kovacs on the "Fine Structural Pathology of the Endocrine System". This chapter, in keeping intellectually and presentationally with the rest of the volume, must be considered a significant contribution to this field.

"Diagnostic Electron Microscopy volume 3", in summary, will provide histopathologists with access to or an interest in electron microscopy with authoritative treatments of several major areas of tumour and non-tumour application: it will be particularly welcomed by those specializing in neoplasia. The price of over £50 cannot be considered high for such a quality volume of over 500 pages.

B. Eden